# A rare case of calcifying fibrous tumor treated with endoscopic full-thickness resection

**DOI:** 10.1055/a-2832-6071

**Published:** 2026-03-30

**Authors:** Xinwei Hao, Fang Liu, Yawei Bi, Jing Yuan, Ningli Chai

**Affiliations:** 1 651943 Department of Gastroenterology, Chinese PLA General Hospital First Medical Center, Beijing, China; 2 651943 Department of Pathology, Chinese PLA General Hospital First Medical Center, Beijing, China

**Fig. 1:**
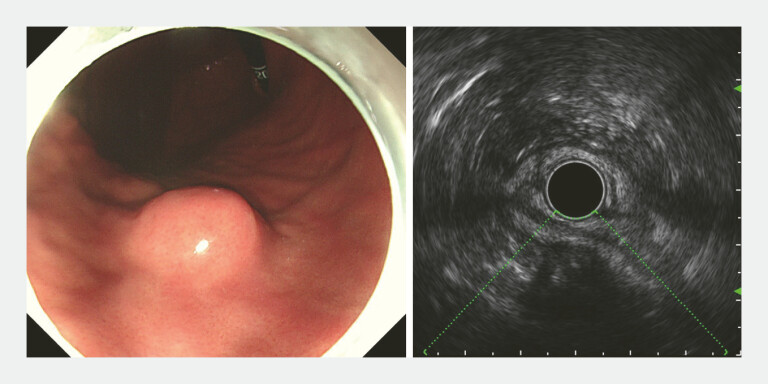
Endoscopic and EUS images. Endoscopy revealed a submucosal tumor on the greater curvature of the gastric body. EUS showed the hypoechoic lesion originated from the muscularis propria layer. EUS, endoscopic ultrasound.

**Fig. 2:**
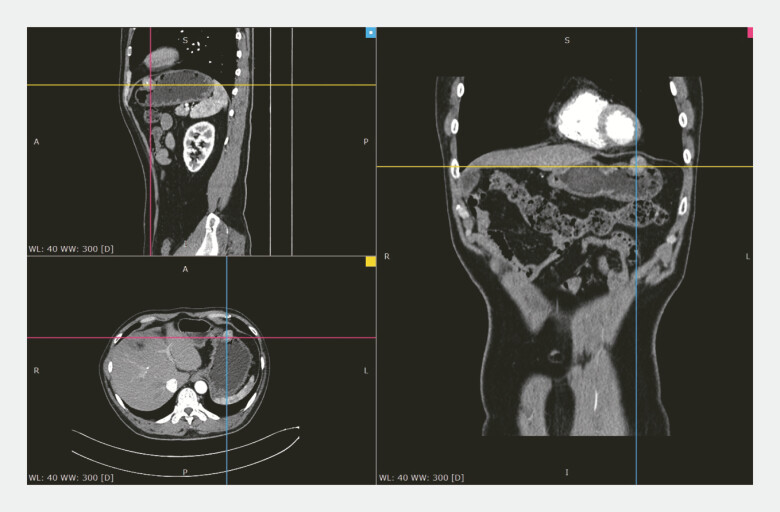
Abdominal enhanced CT showed the lesion arising from the gastric wall was projecting predominantly externally into the abdominal cavity. CT, computed tomography.

A 56-year-old man was referred for further examination after a gastric submucosal mass was discovered. Gastroscopy revealed a 3.0 cm raised lesion on the greater curvature of the gastric body, with a smooth surface. It felt hard to touch with biopsy forceps, and its mobility was somewhat limited. Endoscopic ultrasound showed that the lesion originated from the muscularis propria layer appeared predominantly hypoechoic, with patchy hyperechoic areas in the center. The lesion was exophytic with a clear boundary ( Fig. 1 ). Abdominal enhanced computed tomography showed a round soft tissue mass on the greater curvature of the stomach, measuring approximately 18 × 20 mm. Calcification was visible within the mass, with mild persistent enhancement ( Fig. 2 ).

**Fig. 3:**
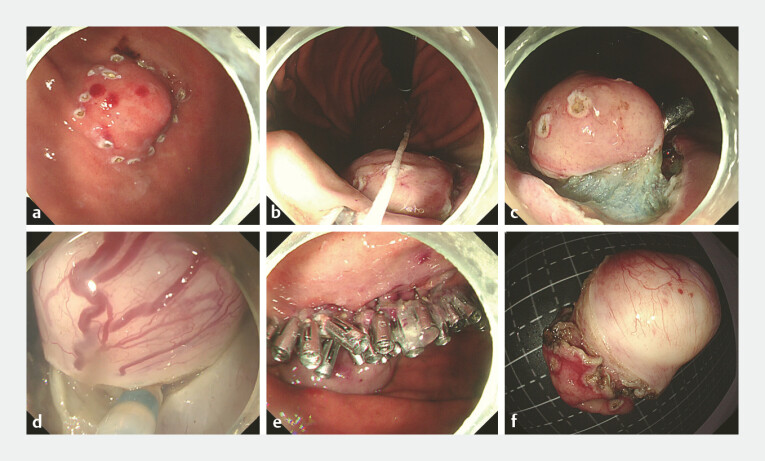
The procedure of endoscopic full-thickness resection. **a** Circumferential marking of the lesion. **b** Traction assistance using a dental floss. **c** Dissecting the lesion along its margins. **d** Continuing dissection after inversion of the lesion with traction. **e** Wound closure with titanium clips. **f** Complete dissection of the lesion.

**Fig. 4:**
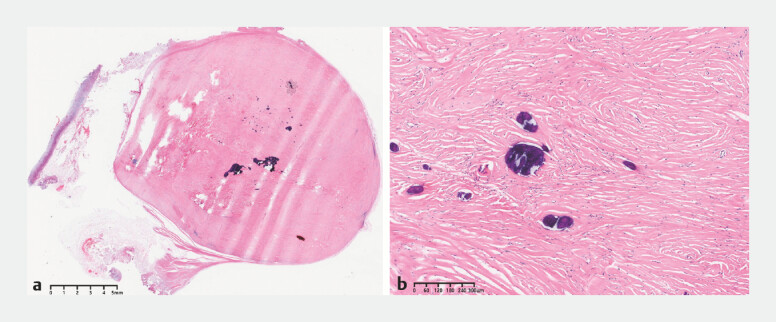
**a, b** Histological features of gastric calcifying fibrous tumor (H&E). H&E, hematoxylin and eosin.

Then, endoscopic full-thickness resection was performed ( Video 1 ). The lesion was gradually dissected with the assistance of a dental floss for traction ( Fig. 3 ). Histologically, the lesion was composed of scant spindle cells embedded in densely hyalinized stroma, with lymphocytic infiltration and multifocal calcification ( Fig. 4 ). Immunohistochemistry demonstrated retained SDHB expression and was negative for CD34, CD117, DOG1, SMA, S100, and PDGFRA. The findings were consistent with a diagnosis of calcifying fibrous tumor (CFT). Postoperatively, the patient was treated with fasting, antibiotics and proton pump inhibitors, leading to an uneventful recovery and discharge.

Endoscopic full-thickness resection for the calcifying fibrous tumor.Video 1

CFT is a rare and benign mesenchymal tumor which can present with nonspecific endoscopic features, often mimicking gastrointestinal stromal tumors 1 2 . In recent years, endoscopic resection has emerged as a feasible option for CFTs. 3 4 We report a case of a gastric CFT with an extraluminal growth pattern that was managed with endoscopic full-thickness resection, achieving the complete resection and closure of the defect.

Endoscopy_UCTN_Code_TTT_1AO_2AG_3AF
